# Real- and *Q*-space travelling: multi-dimensional distribution maps of crystal-lattice strain (∊_044_) and tilt of suspended monolithic silicon nanowire structures

**DOI:** 10.1107/S1600576719015504

**Published:** 2020-02-01

**Authors:** Simone Dolabella, Ruggero Frison, Gilbert A. Chahine, Carsten Richter, Tobias U. Schulli, Zuhal Tasdemir, B. Erdem Alaca, Yusuf Leblebici, Alex Dommann, Antonia Neels

**Affiliations:** a EMPA, Swiss Federal Laboratories for Materials Science and Technology, Center for X-ray Analytics, Dübendorf, Switzerland; b Department of Chemistry, University of Fribourg, Fribourg, Switzerland; c ID01, European Synchrotron Radiation Facility (ESRF), 38043 Grenoble, France; d Universitè Grenoble Alpes, CNRS, Grenoble INP, SIMAP 38000, France; e Department of Mechanical Engineering, Koc University, Sariyer, Istanbul 34450, Turkey; f Surface Science and Technology Center (KUYTAM), Koc University, Sariyer, Istanbul Turkey; g École Polytechnique Fédérale de Lausanne (EPFL), Lausanne, Switzerland; h Sabanci University, Istanbul, Turkey; i ARTORG Center for Biomedical Engineering Research, University of Bern, Bern, Switzerland

**Keywords:** nano-electromechanical systems, NEMS, micro-electromechanical systems, MEMS, nanowires, scanning X-ray diffraction microscopy, lattice tilt and strain mapping

## Abstract

Silicon nanowire-based sensors find many applications in micro- and nano-electromechanical systems, thanks to their unique characteristics of flexibility and strength that emerge at the nanoscale. This work is the first study of this class of micro- and nano-fabricated silicon-based structures adopting the scanning X-ray diffraction microscopy technique for mapping the in-plane crystalline strain (∊_044_) and tilt of a device which includes pillars with suspended nanowires on a substrate. It is shown how the micro- and nanostructures of this new type of nanowire system are influenced by critical steps of the fabrication process, such as electron-beam lithography and deep reactive ion etching. X-ray analysis performed on the 044 reflection shows a very low level of lattice strain (<0.00025 Δ*d*/*d*) but a significant degree of lattice tilt (up to 0.214°). This work imparts new insights into the crystal structure of micro- and nanomaterial-based sensors, and their relationship with critical steps of the fabrication process.

## Abstract 2

A multiscale analytical approach is presented for understanding the structure of micro- and nano-fabricated nanowire-based sensors. Using scanning X-ray diffraction microscopy, crystal deformation is evaluated with respect to lattice strain and tilt caused by the manufacturing process of a silicon monolith.

## 1. Introduction   

In the past few decades, the wide field of applications of electronic devices and the miniaturization of materials have enabled the study and development of devices such as micro- and nano-electromechanical systems (MEMS and NEMS). Thanks to their specific advantages in functionality and performance, nano-electromechanical systems such as sensors, actuators and transistors find application in many sectors including biomedicine (Wu *et al.*, 2018 ▸; Parameswaran *et al.*, 2018 ▸) and aerospace (George, 2003 ▸; You *et al.*, 2018 ▸). However, NEMS often require architectures that involve complex manufacturing and packaging processes that alter the material’s structure by introducing defects, strain and tilt, which, in the long run, influence their performance and can consequently reduce their lifetime dramatically (Dommann & Neels, 2009 ▸). Single-crystalline materials, especially single-crystal silicon (SCSi), are preferentially adopted in MEMS and NEMS technology as they exhibit a high resistance against ageing (Bell *et al.*, 2005 ▸). Reduced lifetime and limited reliability are affected by prestrains and damage introduced by large loads during device manufacture or operation that exceed the critical strength (Dommann *et al.*, 2003 ▸; Neels, Niedermann & Dommann, 2008 ▸). Furthermore, typical manufacturing process steps, such as electron-beam lithography, deep reactive ion etching (DRIE), thermal annealing, dicing and bonding, could favour failure as well (Schifferle *et al.*, 2017 ▸). In addition, such critical steps have a direct impact on the mechanical properties, therefore reducing significantly the lifetime of such devices (Jadaan *et al.*, 2003 ▸). Hence, the characterization of the atomic structure of MEMS and NEMS is extremely important, as very small defects may affect the long-term reliability of devices under working conditions (Fitzgerald *et al.*, 2009 ▸; Neels, Dommann *et al.*, 2008 ▸).

So far, there has been a lot of effort directed towards the characterization of materials behaviour at the nanoscale. However, moving from the micro- to the nanoscale has led to various difficulties in this respect, as the characterization needs to be carried out on the atomic structure to fully understand the relationship between the silicon crystal lattice and the fabrication process. In particular, the atomic structure of SCSi nanowires (NWs) suspended horizontally between two pillars has, to our knowledge, not been studied to evaluate the strain and tilt caused by critical steps of the manufacturing process such as electron-beam lithography and DRIE. We undertake such a study here in order to further our understanding of the crystalline structure of silicon and its physical properties associated with the fabrication processes of MEMS and NEMS and their particular architecture. On the other hand, the characterization of vertical NWs is fairly well known (Kim *et al.*, 2013 ▸; Wallentin *et al.*, 2017 ▸; Diaz *et al.*, 2009 ▸). Suspended Si NWs fabricated by a top-down approach exhibit several advantages, such as a higher yield for batch fabrication (Tasdemir *et al.*, 2016 ▸), the possibility of selective surface application (Yuan *et al.*, 2008 ▸; Baek *et al.*, 2019 ▸), and increased reliability in characterization due to the separation from the substrate and monolithic clamps (Tasdemir *et al.*, 2016 ▸). In addition, the combination of two entities, Si NWs and MEMS, with a three-orders-of-magnitude scale difference, is a direct outcome of this approach, with Arkan *et al.* (2011 ▸) and Nasr Esfahani *et al.* (2017 ▸) demonstrating a mechanism to integrate Si NWs into three-dimensional devices including integrated circuits and MEMS.

To achieve our purpose, investigations by high-resolution X-ray diffraction were performed on a new class of Si NWs, prepared by a new monolithic fabrication approach (Tasdemir *et al.*, 2018 ▸). Although the analysis of the crystal structure of the NW system shows very low strain values and significant levels of lattice tilt, as known from the literature, the capabilities and reliability of MEMS and NEMS often depend on these relatively small variations, which can influence the devices’ ageing behaviour and therefore their lifetime. Monolithically fabricated NEMS–MEMS structures are potentially interesting for applications in space and medicine where a high sensitivity coupled with high reliability is required. In this respect, the analytical approach presented here actively supports the design and fabrication process for such structures, enabling us to take a step forward in understanding the structure of micro- and nano-fabricated SCSi NW-based sensors.

## 2. Sample fabrication and description   

**Figure 1:**
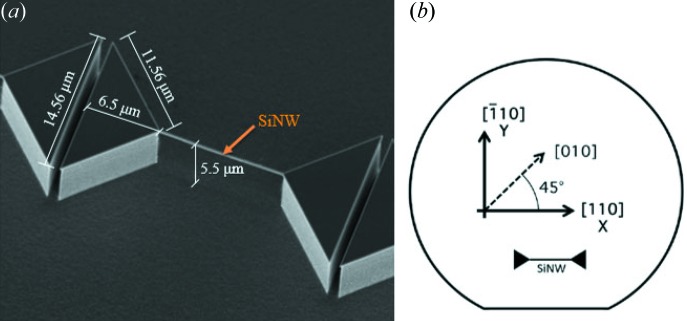
(*a*) An SEM image showing the sample dimensions. (*b*) A schematic representation of the crystallographic directions of the monolithic Si nanowire (SiNW) system.

The samples used in this work were fabricated by etching a thick silicon substrate (〈100〉 Si wafer about 500 µm thick) with the help of top-down micro/nano-fabrication methods (Fig. 1 ▸). The fabrication process begins with a nano-patterning step through electron-beam lithography, using an inorganic negative-tone resist called hydrogen silsequioxane (HSQ), where both the linewidth and length of the NWs are defined on the wafer. A shallow reactive ion etching follows, where a surface protrusion is obtained. Then, low-temperature oxide is coated on the side walls of the protrusion, protecting it during the upcoming deep etch step, where an undercut releases the protrusion in the form of a suspended NW. These NWs are suspended at a distance of 5.5 µm from the substrate beneath. Triangular pillars at both ends of the NWs are used to keep the NWs attached to the substrate. Further details regarding the fabrication process are explained elsewhere (Tasdemir *et al.*, 2016 ▸, 2018 ▸). Although the bulk-scale (millimetre- and even micrometre-scale) properties of silicon have been studied in great detail (Gaither *et al.*, 2010 ▸, 2011 ▸; Schifferle *et al.*, 2017 ▸), the nanoscale properties of silicon still remain an interesting matter of investigation. Here, we focus on the identification of the nanometre-scale intrinsic properties of silicon, possibly coming from the fabrication processes, using high-resolution X-ray diffraction.

It is well known that the interaction between an energetic electron beam and a material produces various types of damage such as heating and displacement due to the elastic and inelastic scattering of electrons (Egerton *et al.*, 2006 ▸; Chang, 1975 ▸; Jackel *et al.*, 1984 ▸). However, in our case electron-beam lithography was just used as the first step to pattern the resist layer (HSQ) for the definition of the NW width and the pillars. Therefore, the strain and tilt introduced in the crystal lattice are mainly due to deep reactive silicon ion etching (DRIE) (Oehrlein, 1989 ▸; Pang *et al.*, 1983 ▸; Oehrlein *et al.*, 1984 ▸). The combination of very different scales in one device, such as micrometre- and nanometre-sized parts in MEMS and Si NWs, respectively, has always been the bottleneck for a single-step manufacturing process. In this regard, a unique monolithic process based on an improvement of the SCREAM process (single-crystal reactive etching and metallization; Shaw *et al.*, 1994 ▸) was developed for the fabrication of a single NW within a thick silicon layer. Thanks to this approach, the manufacturing limits of batch fabrication for NEMS/MEMS processed from the same crystal can be overcome, leaving the surface accessible for further surface processing such as doping and/or electrical contact formation. A detailed description of this process is given by Tasdemir *et al.* (2018 ▸).

## 3. Scanning X-ray diffraction microscopy on ID01   

To investigate the strain and tilt of the silicon crystal lattice in a given device architecture, X-ray diffraction (XRD) analysis is used as the most suitable technique. Although methods such as electron diffraction or high-resolution transmission electron microscopy provide atomic resolution, they inherently have a poor penetration depth due to the strong interaction between electrons. Hence, their use is limited to the investigation of small regions in the sample without providing an overall view of the material as can be done using XRD. Using a nano-focused X-ray beam, we can penetrate sufficiently into the structure, mapping the strain and tilt distribution inside the crystal lattice of the whole NW system. High-resolution XRD is a non-contact and non-destructive analytical method that is ideal for electronics, optoelectronics, X-ray optics and sensors based on semiconductor materials (Rait & Peiser, 1957 ▸; Hanke *et al.*, 2008 ▸; Epp, 2016 ▸). In this study, the analytical investigation was performed by means of scanning X-ray diffraction microscopy (SXDM). This non-destructive method combines high-resolution spatial scanning with reciprocal-space mapping, allowing information on lattice strain and tilt to be extracted (MacDowell *et al.*, 2001 ▸; Meduňa *et al.*, 2018 ▸). This technique can also be adopted using either a monochromatic or a polychromatic (white) beam, according to the sample type and size with respect to the dimensions of the incoming beam (Tamura *et al.*, 2005 ▸, 2009 ▸).

Related to the recent progress in X-ray sources, focusing optics and fast two-dimensional area detectors, it is also possible to perform different *in situ* analysis under mechanical and thermal stress on a wide range of different materials (Tamura *et al.*, 2003 ▸; Tippabhotla *et al.*, 2017 ▸). The high spatial resolution of between 200 and 800 nm on beamline ID01 at the European Synchrotron Radiation Facility (ESRF, Grenoble, France) provides a high strain sensitivity below Δ*d*/*d* = 10^−5^ and a resolution of the variation in the degree of tilt down to 10^−3^, and is therefore ideal for our investigations (Chahine *et al.*, 2014 ▸). This method is often called K-map (quick mapping) as it works with continuous motion for a faster and more efficient collection of data.

The data evaluation is associated with the analysis of the Bragg-peak displacement and shape seen in three-dimensional reciprocal-space maps (3D-RSMs). By collecting thousands of two-dimensional detector images at different incident angles (ω), we built 3D-RSMs of the 044 Bragg reflection at each sample position. In the Bragg condition, we analysed the variations in the scattering vector **Q** passing through the sample [*Q* = (4π/λ)sin(θ/2), where θ is the scattering angle and λ is the wavelength of the incident radiation], measuring changes in length (lattice strain) and angular deviation (lattice rotation) of the **Q** components (*Q_x_*, *Q_y_* and *Q_z_*) from an orientation defined as a reference on a two-dimensional detector (Kriegner *et al.*, 2013 ▸). We first generate two-dimensional plots of the intensity distribution by linking and merging the detector images to their *y*, *z* motor positions. Then, in order to build 3D-RSMs, the conversion from real- to reciprocal-space coordinates (both Cartesian and angular) is possible through the use of rotational matrices which link and merge together two-dimensional detector images taken at different motor positions (*y* and *z*) and different incident angles (ϕ). This is done directly in the software once the values of the parameters (direct beam position, degrees per detector angle *etc.*) have been entered. By doing so, we can efficiently separate lattice information such as strain and tilt.

Considering the potential applications of such devices, it is necessary to analyse all of the architectural features, such as the NW itself and the triangular pillar support structure, as this will later be used for NW actuation and/or readout modes. We used a versatile method allowing us to analyse simultaneously all multiscale and multi-architecture regions of the sample: the pillars with micrometre dimensions on a millimetre-sized substrate and the NW. The use of a nanobeam and other experimental details mentioned in the next section were of fundamental importance in achieving our goal.

### 3.1. Experimental setup   

**Figure 2:**
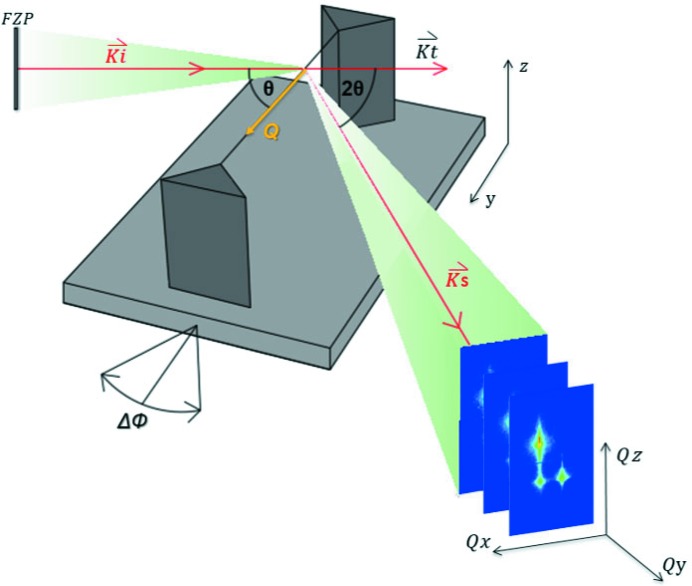
A sketch of the experimental setup for a horizontal Bragg diffraction configuration. Scans were performed by moving the sample along the *y*- and *z*-axis directions. These were repeated after varying the incidence angle ϕ in steps of 0.007°, while keeping the detector fixed at an angle corresponding to the 044 Bragg reflection at 2θ = 107.63°. **K**_i_ is the incident wavevector, **K**_t_ is the transmitted vector and **K**_s_ is the scattered vector. *Q_x_*, *Q_y_* and *Q_z_* are the coordinates in reciprocal space.

SXDM was performed using an 8 keV beam focused down to 350 nm (horizontal) × 200 nm (vertical) by means of a Fresnel zone plate (FZP) with a 300 µm diameter and an 80 nm outer zone width. A 50 µm molybdenum pinhole was mounted downstream of the FZP to filter out higher diffraction orders. The sample was mounted on a high-precision piezoelectric scanning stage (*x*, *y*, *z*), which was used to move the sample with respect to the incoming beam. In addition, an optical microscope was mounted above the sample for quick alignment during scans. A two-dimensional MAXIPIX photon-counting detector was used to collect images of 516 × 516 pixels at each *y*, *z* scan position. According to the shape of the sample, we opted for a horizontal Bragg diffraction configuration in order to investigate the in-plane strain (∊_044_) in the whole NW system, which allows efficient decoupling of the NW and pillar information from the substrate. After satisfying the experimental geometric Bragg condition to detect the 044 reflection at 2θ = 107.63° (Fig. 2 ▸), we started alignment by shooting the substrate and moving the whole system slowly downwards, along the *z*-axis direction, until we found the diffraction peak coming from the NW, which is anchored at the top surface of the pillars. We then scanned the NW following its long axis, along the direction of the *y* axis. Once we arrived at the pillar and detected its signal, we moved the sample upwards along the *z* direction, scanning the sidewall of the pillar. In addition, in order to build 3D-RSMs we performed a rocking scan at every *y*, *z* point, varying the ϕ angle in steps of 0.007° around the 044 Bragg reflection. In this way, we collected thousands of two-dimensional diffraction patterns, which allowed us to check the scattering conditions in the vicinity of the 044 peak and build 3D-RSMs.

## 4. Results and discussion   

**Figure 3:**
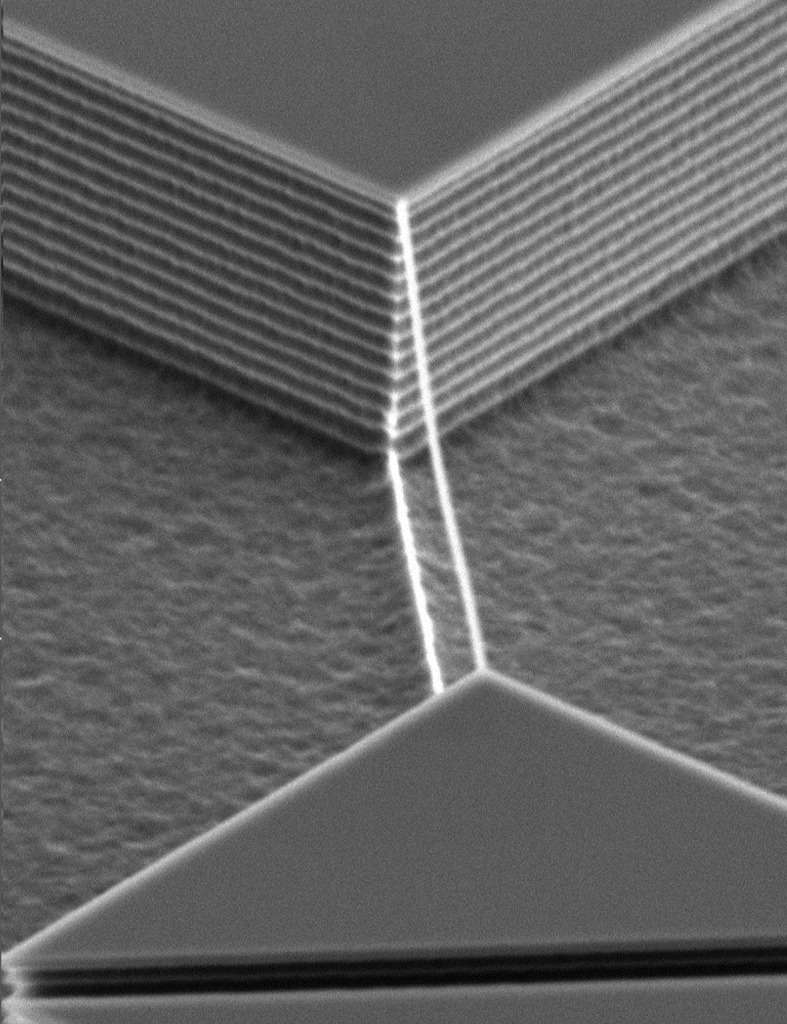
The scallops on the pillar side walls generated during DRIE.

The DRIE process results in changes in the crystal structure because of the reaction between chemically reactive species and electrically charged particles (Laermer *et al.*, 2015 ▸). The positively charged cations generated in this manner are accelerated towards the substrate, promoting the chemical reaction and inducing variations in the electronic clouds of the atoms constituting the crystal structure. Also, since the etching depth increases progressively step by step, a significant degree of tilt is introduced into the structure, especially between the top surface of the pillars and the underlying substrate. At the end of each step, the anisotropy of etching decreases because of the excessive formation of etchant species, leading to roughness formation, also known as scalloping, on the side walls of the pillars (Fig. 3 ▸), although this did not affect the present analysis.

### 4.1. Correlation of 3D-RSMs and 2D-RSMs   

**Figure 4:**
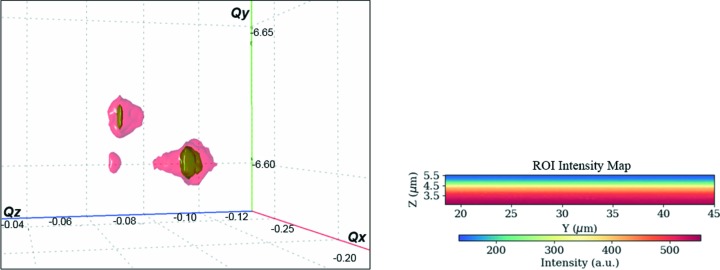
A three-dimensional representation of the Si 044 Bragg peaks in *Q* space for a given real-space position (*y* = 21.5, *z* = 5) on the ROI intensity map.

Tracing the intensity distribution in real-space images for selected regions of interest (ROIs) in reciprocal space is an initial stage of qualitative analysis on the state of the sample crystal structure. An inhomogeneous distribution of the integrated intensity over a certain scanned region indicates the presence of structural deviations from a hypothetical perfection of the crystal, such as the presence of defects, strain and tilt distribution, degree of mosaicity *etc*. However, for an appropriate and detailed analysis it is necessary to investigate a Bragg-peak position at each scanned point of the sample. For this purpose, we recorded two-dimensional diffraction images every 300 nm along the *y* axis and every 120 nm along the *z* axis for different incident angles with steps of 0.007° in a range of 1.5° centred on the Bragg position, thus generating 3D-RSMs at each sample position. Hence, we end up with five-dimensional data to process and analyse: two dimensions for real-space positions *y* and *z*, one for the incident angle θ, and two for the detector scattering angles defined by 2θ and the deviation angle ν (the distance between the centre of the detector and the recorded signal on the detector). In this way, we define the reference values of the scattering vector *Q*(*xyz*) on the detector. The data treatment, by fitting and parsing two-dimensional real-space images of the diffracted intensities with 3D-RSMs, was performed using the *X-ray Strain Orientation Calculation Software* (*XSOCS*) recently developed on the ID01 beamline at the ESRF (Chahine *et al.*, 2014 ▸). The *XSOCS* software goes through all the recorded frames by matching the peak intensity values recorded at each *y*, *z* position of the sample, and as a result converts the scattering angles into Cartesian coordinates (*Q_x_*, *Q_y_*, *Q_z_*) or angular coordinates (roll, pitch and radial) (Kriegner *et al.*, 2013 ▸). This allows one to work simultaneously in both real and reciprocal space, visualizing the evolution in shape and position of the three-dimensional Bragg peaks at each position in a two-dimensional real-space map (Fig. 4 ▸) (see also movies 1–3 in the supporting information).

**Figure 5:**
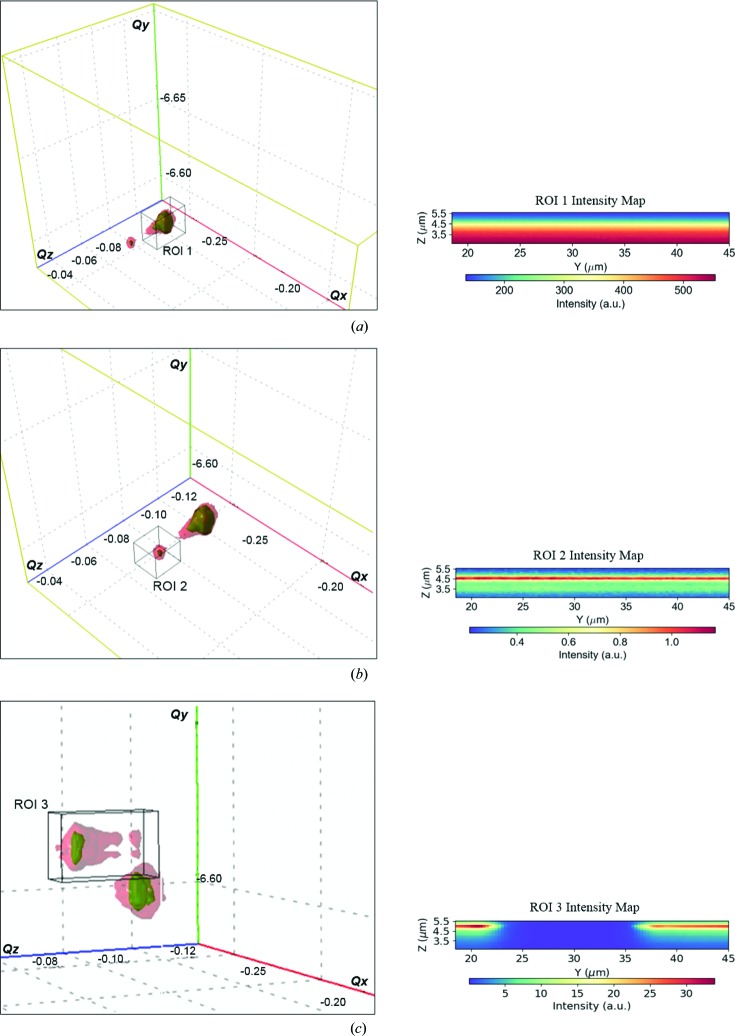
Two-dimensional real-space maps obtained by integrating the diffracted intensity over the regions (*a*) ROI 1, (*b*) ROI 2 and (*c*) ROI 3 in the 3D-RSM.

The presence of three 044 Si Bragg peaks at the same time in different positions in *Q* space is related to specific regions of the sample, such as the vertex of the pillars where the NWs are anchored (*Z* = 5.5 µm). This is mainly due to the combination of several factors like the sample shape, the scattering geometry, the scan mode (Map + RCs), and the divergence of the incident and diffracted beams. However, once the sample is fixed at the centre of rotation, the occurrence of the 044 Si reflection simultaneously in different *Q* positions is only possible if the different regions of the sample (substrate, pillars and NWs) are affected by lattice deformation. Otherwise, since the sample is entirely made of crystalline silicon, we would have had the silicon Bragg peak always at the same *Q* position, which is the reference position of the 044 silicon reflection (zero strain/tilt). However, such physical phenomena are quite useful for separating information from different regions of the sample. Additionally, by integrating the intensities over a specific ROI of the RSM (Fig. 5 ▸), two-dimensional real-space maps of the intensity can be updated to analyse the trend of their distribution over a specific region of the sample. Any variations in shape and displacement of the 044 silicon Bragg peak in *Q* space for different sample positions indicate the presence of high strain and/or tilts (°) of the crystal lattice induced by the DRIE. This can be seen from any deviation with respect to the reference main peak inside ROI 1, as shown in Figs. 5 ▸(*b*) and 5 ▸(*c*). The reference peak inside ROI 1 was taken by scanning from the surface of the substrate, and thus from the bottom of the pillars to their upper surface. By integrating the intensity on ROI 1 as shown in Fig. 5 ▸(*a*), we see a gradient in the spatial distribution of the diffracted intensity, which increases when moving from the upper part of the wall and approaching the bottom of the pillars. This may indicate the presence of tilt and/or strain contrast within the pillars, and between the pillars, the NW and the substrate. By integrating the intensities over ROI 2 [Fig. 5 ▸(*b*)], we see a horizontal line in the real-space map indicating the region contributing to this diffraction peak. This is the NW of roughly 12 µm length. Such a horizontal line appears about 1 µm away from the top of the pillar surface when approaching the middle of the NW. Also, when approaching the top surface of the pillar’s side wall, a third peak (ROI 3) with a weak surrounding diffuse scattering [Fig. 5 ▸(*c*)] arises in a different *Q*(*xyz*) position, thus showing the presence of lattice strain and tilt.

**Figure 6:**
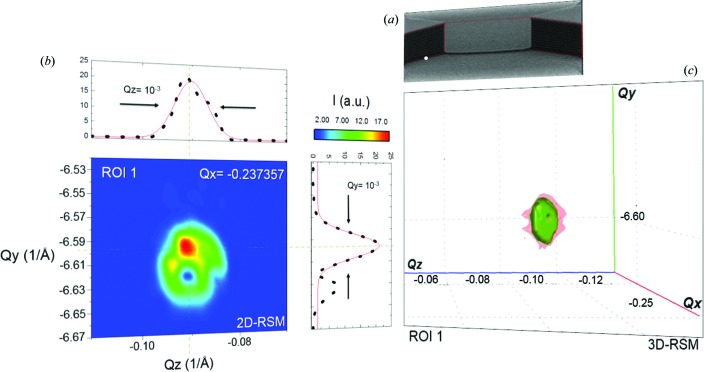
Reference analysis. (*a*) A SEM image, with the white dot representing one of the many scanned points that contribute to the reference peak within ROI 1. This point, located at the base of the pillar, is also used as a reference for the tilt and strain calculations, since they are directly attached to the substrate. (*b*) 2D- and (*c*) 3D-RSMs of the Si 044 reflection recorded at different incident angles. The Si 044 peak is plotted as an isosurface. The measured 1D patterns (dots) are fitted (red curve) with the Gaussian function *y* = *y*_0_ + [*A*/*w*(π/2)^1/2^] exp{−2[(*x* − *x*_c_)/*w*]^2^}. Further details are provided in the supporting information.

Since the device is entirely made of single-crystal silicon, any scanned part on the sample unaffected by a certain level of strain and tilt will contribute to the peak within ROI 1 at *Q_x_* = −0.237, *Q_y_* = −6.590 and *Q_z_* = −0.090 in reciprocal space. Figs. 6 ▸, 7 ▸ and 8 ▸ show some of the scanned points for ROIs 1, 2 and 3 with their most significant 2D- and 3D-RSMs. The white point in Fig. 6 ▸(*a*) represents one of the many and most significant scanned points contributing to ROI 1. This point is also taken as a reference for the tilt and strain calculations and map generation. The projection *Q_y_*–*Q_z_* corresponding to the Si 044 reflection in Figs. 6 ▸(*b*) and 6 ▸(*c*) reveals a sharp peak stemming from the strain- and defect-free state. In particular, such a peak depicts roughly the shape of the optical devices, which also limits the peak width. This is essentially due to the FZP and to the angular size of the detector pixels, which induce divergences of ∼0.08 and ∼0.003°, respectively. Such a physical effect also influences the fitting curve (red line) on the *Q_y_* projection in Fig. 6 ▸(*b*), which does not match the measured data perfectly. Additional details are included in the supporting information.

**Figure 7:**
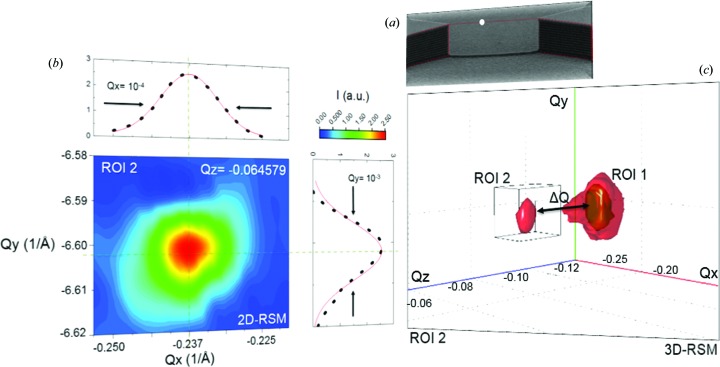
NW analysis. (*a*) A SEM image, with the white point indicating the scanned point taken near the centre of the NW. (*b*) A *Q_x_*–*Q_y_* projection with a 1D profile, obtained from (*c*) the 3D-RSM around the Si 044 reciprocal-lattice points.

The scans recorded along the NW show a small peak in a new position in *Q* space. One of these scanned points is shown on the scanning electron microscopy (SEM) image in Fig. 7 ▸(*a*). The white circle indicating the scanned point is located at *Q_x_* = −0.2375, *Q_y_* = −6.6015 and *Q_z_* = −0.0645 in *Q* space. In this case, the occurrence of an extra Si 044 peak in reciprocal space indicates the presence of lattice tilt and/or strain between the NW and the bulk, consisting of the supporting pillars and the underlying substrate. Physical phenomena such as lattice deformations are very often derived from the combination of multiple defects within the atomic structure, such as screw and line dislocations, vacancies, atomic substitutions and so on. However, this was not an object of the present study.

**Figure 8:**
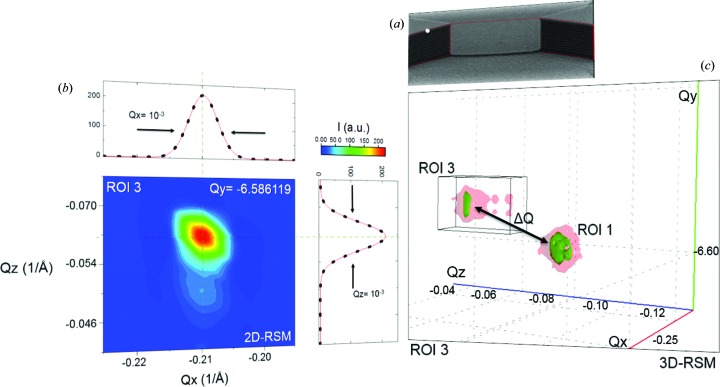
The edge of the pillars. (*a*) A SEM image, with the white dot indicating one of the measured points. (*b*) A 2D-RSM with 1D sections *Q_x_* and *Q_z_* obtained from (*c*) the 3D-RSM. The 2D-RSM is plotted as an isosurface. The measured 1D patterns (dots) are fitted (red curve) with the Gaussian function *y* = *y*_0_ + [*A*/*w*(π/2)^1/2^] exp{−2[(*x* − *x*_c_)/*w*]^2^}. Further details are provided in the supporting information.

During the mapping of the pillars from the bottom to their top part, we recorded a new position of the Si 044 peak in *Q* space (ROI 3) at *Q_x_* = −0.21, *Q_y_* = −6.58 and *Q_z_* = −0.060. This occurs with the increasing height of the pillars, mainly from the middle (*z* = 2.75 µm) to the top of the pillar walls (*z* = 5.5 µm) (Fig. 8 ▸). According to the diffracted intensity distribution, which varies in the vertical direction, one would also expect similar changes in the Bragg peaks in the same direction, *i.e.* from the bottom to the end of the pillar wall. The appearance of a new peak in a different *Q* position indicates once again the presence of a strong degree of tilt between the pillars and the substrate on which they are anchored. The quantification of Δ*Q* to confirm whether the sample is affected by strain and/or tilt is given in the next section. The small amount of diffuse scattering around the 044 reflection within ROI 3 [Fig. 8 ▸(*c*)], characterized by fringes, is also visible in Fig. 8 ▸(*b*) along the *Q_z_* direction. This occurs only when one approaches the upper surface of the pillar’s side wall, indicating an increase in defects in that region of the device.

The quality of NEMS- and SiNW-based sensors is also linked to the degree of crystal perfection, which in turn is related to the peak shape and intensity. A decrease in crystalline order results in a symmetrical broadening together with a decrease in the intensity. On the other hand, if the crystal lattice is affected by deformation, the strain profile would increase the asymmetry due to the direction dependence of the strain. As we have seen so far from the analysis of Bragg peaks for a given sample position, the Si 044 reflection does not show any significant changes in the peak width. Certainly, the peak shape changes according to the volume probed by the X-ray beam. The measurement clearly shows new positions of the 044 Si peak in reciprocal space, thus indicating a significant degree of inclination between the NW, the pillars and the substrate.

### 4.2. Construction of two-dimensional distribution maps of lattice strain   

**Figure 9:**
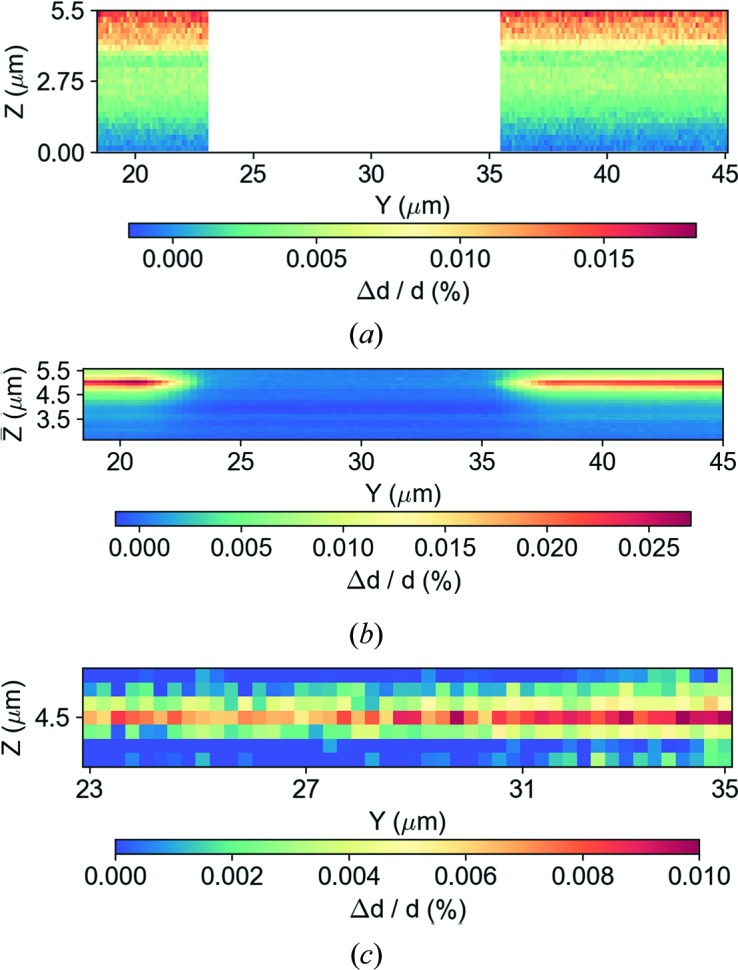
Real-space distribution maps of in-plane strain (∊_044_) as a function of the beam position on the sample. (*a*) A strain plot generated from the reference 044 silicon main peak within ROI 1. (*b*) A strain plot obtained from the *Q*(*x*, *y*, *z*) values of the peak within ROI 3 and (*c*) a strain plot from the peak inside ROI 2.

The strain of the crystal lattice is strictly connected to the variation in the *d* spacing, which in turn implies changes in the scattering vector **Q**. The *XSOCS* software was used to perform a complete characterization of these physical parameters. As described above, this is possible after resampling the measured detector images in *Q* space for each beam position (*y*, *z*) on the sample, with **Q** = **K**_s_ − **K**_i_, where **K**_s_ and **K**_i_ are the scattered and incident wavevectors, respectively. The strain ∊_*hkl*_ is the projection of the scattering vector **Q** and describes the deviation of the *d* spacing and, inversely, of the length of the scattering vector:
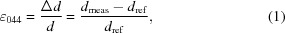
where *d*_ref_ is the reference *d* spacing of unstrained crystalline silicon (*d* = 0.95365 Å) taken in the middle of the pillar bulk at *z* = 2.75 µm and *y* = 18 µm, and *d*_meas_ is derived from the measurements:
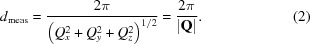
All scanned points of the sample contributing to the Bragg peak within ROI 1 are considered as the unstrained state, and any variation in the position and shape of that peak in *Q* space will be the result of a variation in the *d* spacing and therefore in the lattice strain. As we would expect from the intensity distribution and from the different positions of the 044 Bragg peak in *Q* space, the in-plane strain (∊_044_) distribution map in Fig. 9 ▸(*a*) shows Δ*d*/*d* (%) distributed differently, mostly along the pillar height. The presence of a peak within ROI 3 shows the top of the pillar wall reaching strain values up to 0.025% [Fig. 9 ▸(*b*)]. Furthermore, between scanned points 4 and 8, near the centre of the NW [Fig. 9 ▸(*c*)], the strain values vary between 0.008 and 0.0175% due to the presence of a 044 Bragg peak in a different *Q* position (ROI 2). Although the fluctuations in these values are quite small, thanks to the high sensitivity of the method the measurements still produce a significant contrast, proving that the NW is slightly relaxed with respect to the upper surface of the pillar side wall. This might be due to a tilt between the pillars, which causes a slight deviation of the NW direction.

### 4.3. Two-dimensional distribution of lattice tilt   

**Figure 10:**
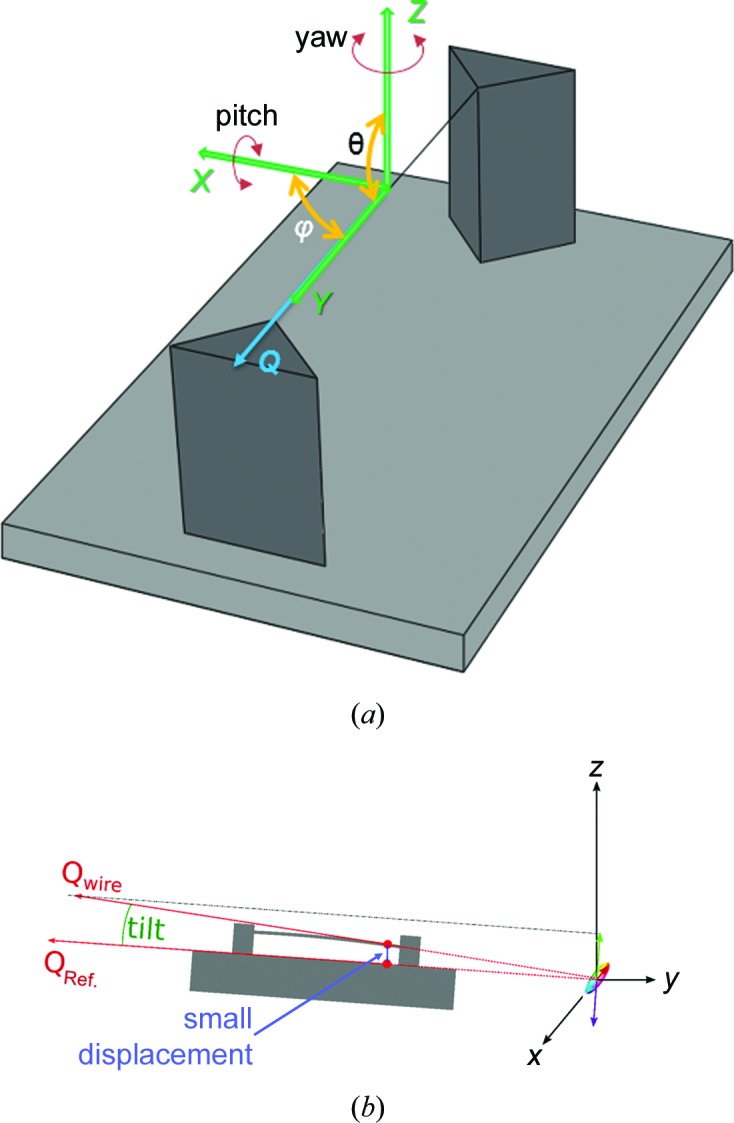
(*a*) A schematic representation of the spherical coordinates roll, pitch and yaw related to the projection of the scattering vector **Q** and the sample position in the *xyz* coordinate system. (*b*) A sketch of a pitch rotation which, given two points at different *z* positions, involves an angular deviation (θ) between the *z* axis and *Q_z_*. A rotation about the *z* axis instead (yaw) will result in an angular difference (φ) between the *y* axis and the vector component *Q_y_*.

The lattice tilt was determined with respect to the bottom of the pillars attached to the upper surface of the substrate (*y* = 42.1173 µm and *z* = 0 µm), by converting the Cartesian coordinates into spherical ones (see supporting information). Each coordinate *x*, *y*, *z* is associated with a rotation about its axis (roll, pitch and yaw), also commonly known as Eulerian coordinates. Using this notation, it is possible to describe for a rigid body any rotation around three orthogonal axes in order to define a new position of the body in space. However, our spherical coordinates representation, according to the functionality of the piezoelectric sample stage and the experimental geometry, is sensitive to rotations about the *x* and *z* axes, pitch and yaw, respectively [Fig. 10 ▸(*a*)]. As can be seen from the figure, a pitch rotation induces a variation in the θ angle between the *y* and *z* axes, or the angle between the projection of the **Q** vector on the surface of the sample and the *z* axis. On the other hand, yaw involves a rotation about the *z* axis, implying changes in the φ angle, or the angle between the *x* axis and the projection of the **Q** vector coinciding with the *y* axis.

**Figure 11:**
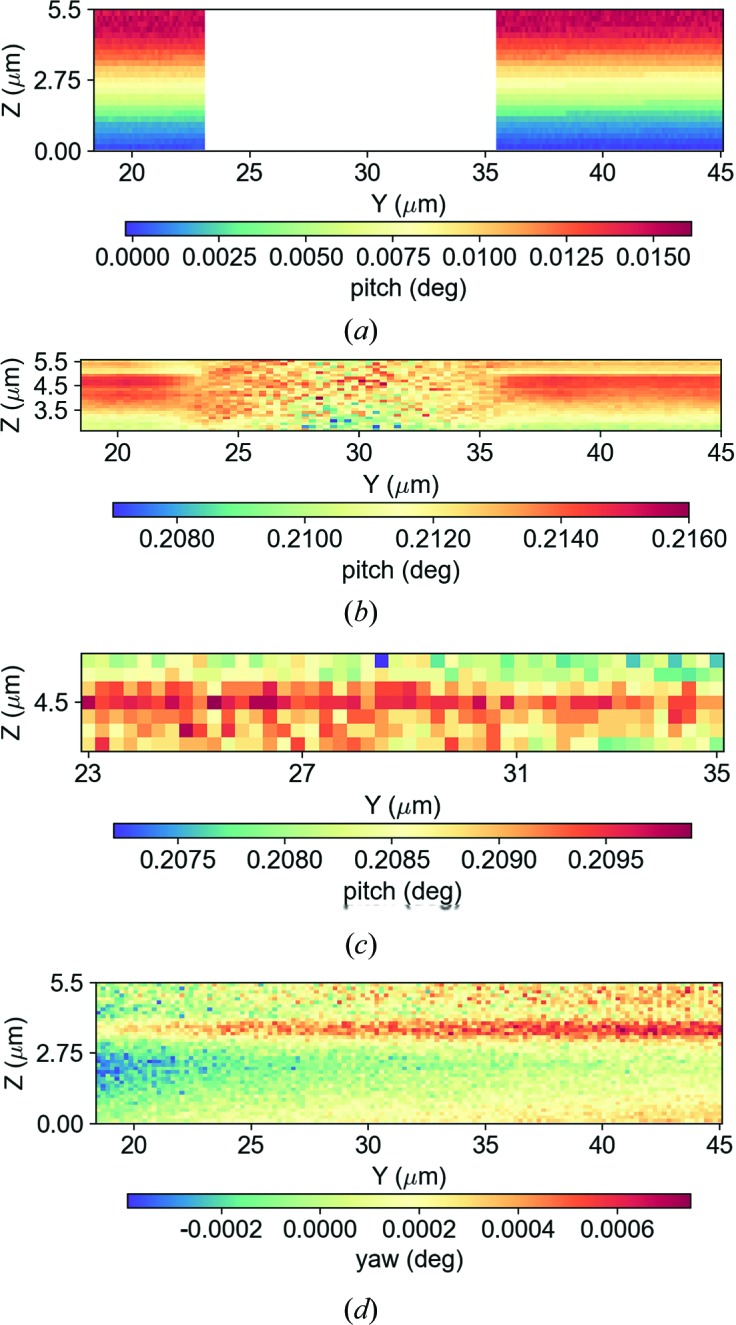
Two-dimensional distribution maps of the magnitude of the degree of tilt. Plots (*a*) generated from the reference peak within ROI 1, (*b*) related to ROI 3, and (*c*) and (*d*) related to ROI 2.

A proper quantification can be done by extracting all the *Q*(*xyz*) values for each sample position and then using the following mathematical relation:
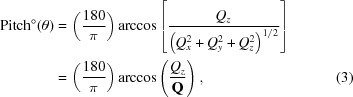


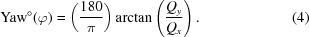
In Fig. 11 ▸, the shift away from the zero positions of the angular coordinates of the scattering vector **Q** indicates that the sample is not only affected by strain, but mainly shows a significant degree of tilt of the crystal lattice, in both NWs and pillars, with respect to the underlying substrate. Fig. 11 ▸(*a*) shows the gradient of the degree of tilt increasing with the height of the pillars, with no tilt at the bottom of the pillar and rising up to 0.016° at the upper surface of the latter. In particular, the presence of a peak within ROI 3 is explained by the significant degree of tilt of the upper part of the pillar side wall at *z* = 5.5 µm, reaching values up to 0.2140° [Fig. 11 ▸(*b*)], with the scattering vector **Q** pointing upwards with respect to the base, as shown in Fig. 10 ▸. This is also consistent with the splitting of the peak at the top of the pillar. Instead, the peak within ROI 2 [Fig. 11 ▸(*c*)] shows that a large part of the NW is tilted with respect to the bottom of the pillar, reaching values up to 0.2095°. In addition, in the vicinity of the scanned point number 6, the NW is approximately 1 µm lower than the upper surface of the pillar wall, the spot where the NW is anchored. This may be due to the tilt direction of the pillars arising during the various steps of the deep silicon etching process. Furthermore, the angular discrepancy in φ, due to the deviation of the **Q** vector from the *y* axis, shows a yaw tilt degree much lower than pitch, reaching values up to 0.0006° along the NW [Fig. 11 ▸(*d*)]. Such physical conditions attest to a good alignment between two adjacent pillars.

## 5. Conclusions   

A new class of silicon NWs suspended between two triangular pillars, obtained from the same thick silicon crystal, has been investigated. The combination of three-dimensional reciprocal-space maps and two-dimensional real-space distribution maps allowed us to make a full analysis of the in-plane strain (∊_044_) and tilt (°) distribution. The structural features discussed herein are derived from the NEMS design and the related critical steps of the fabrication process, such as electron-beam lithography and deep reactive ion etching. Through measurements by means of scanning X-ray diffraction microscopy, we were able to access the structural properties of the silicon-based suspended NWs. The large data sets for this monolithic system were handled for more than three dimensions [(*y*, *z*) for scanning real-space positions, θ for the incident angle, and 2θ and ν for the detector scattering angles], thus separating the important structural information [the length of the scattering vector (lattice strain) and its angular deviation (lattice rotation)].

Our results show that deep silicon etching is the main critical fabrication step generating slight changes in the 044 Bragg reflection, and is therefore responsible for the variation in the crystal lattice deformation. As we expected, the strain distribution introduced in the NW has a very small value. The fluctuation appears in the third and fourth decimal places, reaching maximum values of 0.00011 (Δ*d*/*d*) along the NW and 0.00025 for the upper surface of the pillar side wall. Nevertheless, given the high sensitivity of the method, the measurements still produce a significant contrast, showing that the NW is slightly less strained than the pillar’s upper surface. Furthermore, sequential etching in the top-down approach introduces an important tilt (°) magnitude in the crystal lattice, visible from the appearance of two extra peaks at different positions in reciprocal space (ROI 2 and ROI 3). At about each micrometre etched on the silicon wafer, the lattice tilt of the sample increased by 0.0389°, reaching a maximum value of 0.214° at the top surface of the pillar’s side wall. The same pitch tilt magnitude is present and distributed differently along the entire NW, with fluctuations between 0.2090 and 0.2095°. Although lattice strain and tilt may not necessarily be connected to each other, in this case the only clear correlation between them is visible in a gradient that increases from the lower part of the pillars to their upper surface located 5.5 µm from the substrate. Furthermore, the centre of the NW appears roughly 1 µm lower than its anchor points. Additionally, we do not see any particular correlation between strain and the tilt distribution along the NW.

In MEMS and NEMS devices, the evaluation of the strain and tilt of a crystal lattice is essential as it is also linked to the defect state of the material, and therefore to the quality of the device. The collected data show some weak diffuse scattering around the analysed Bragg reflection, indicating that defects may be present in the machined silicon structure. However, surface defects are also subject to recombination and would move out of the silicon over time. The investigation of the impact of defects and related effects on device functioning will be the subject of a future study.

The overall device structure contributes significantly to the long-term operation of that device. With this study, we have started to address the high demand for quality and reliability coupled with enormous analytical challenges, which are particularly true for nano-sized sensors probing small effects within foreseen space or medical applications.

## Supplementary Material

Movie 1 (ROI 2 selection). DOI: 10.1107/S1600576719015504/ks5638sup1.mp4

Movie 2 (ROI 3 selection). DOI: 10.1107/S1600576719015504/ks5638sup2.mp4

Movie 3 (making projections). DOI: 10.1107/S1600576719015504/ks5638sup3.mp4

Additional tables and figures. DOI: 10.1107/S1600576719015504/ks5638sup4.pdf
