# Supplementary material for: Real- and Q-space travelling: multi-dimensional distribution maps of crystal-lattice strain (∊044) and tilt of suspended monolithic silicon nanowire structures
Source: J Appl Crystallogr. 2020 Feb 1;53(Pt 1):58–68. doi: 10.1107/S1600576719015504 (PMC6998783; doi:10.1107/S1600576719015504)

JOURNAL OF
APPLIED
CRYSTALLOGRAPHY

Volume 53 (2020)

Supporting information for article:

Real- and *Q*-space travelling: multi-dimensional distribution maps of crystal-lattice strain (ε_{044}) and tilt of suspended monolithic silicon nanowire structures

Simone Dolabella, Ruggero Frison, Gilbert A. Chahine, Carsten Richter, Tobias Schulli, Zuhal Tasdemir, B. Erdem Alaca, Yusuf Leblebici, Alex Dommann and Antonia Neels

Table S1

Model	Gauss
Equation	y=y0 + (A/(w*sqrt(pi/2)))*exp(-2*((x-xc)/w)^2)
Plot	Qy (ROI 1)
y0ffset	-2.24369E-4 ± 0.0276
xcenter	-6.59705 ± 2.08869E-4
widht	0.00463 ± 4.43981E-4
Area	0.00762 ± 6.82713E-4
Reduced Chi-Sqr	0.07029
R-Square (COD)	0.64151
Adj. R-Square	0.63224

Model	Gauss
Equation	y=y0 + (A/(w*sqrt(pi/2)))*exp(-2*((x-xc)/w)^2)
Plot	Qz (ROI 1)
y0ffset	-0.00318 ± 0.03057
xcenter	-0.08847 ± 1.4772E-4
widht	0.00336 ± 3.10169E-4
Area	0.00633 ± 5.5169E-4
Reduced Chi-Sqr	0.08684
R-Square (COD)	0.64782
Adj. R-Square	0.63887

Table S2

Model	Gauss
Equation	y=y0 + (A/(w*sqrt(pi/2)))*exp(-2*((x-xc)/w)^2)
Plot	Qx (ROI 2)
y0	0.21787 ± 0.16139
xc	3335.90548 ± 0
w	8.03238E-4 ± 0
A	0.00952 ± 0
Reduced Chi-Sqr	2.96931
R-Square (COD)	-1.11022E-15
Adj. R-Square	-0.02727

Model	Gauss
Equation	y=y0 + (A/(w*sqrt(pi/2)))*exp(-2*((x-xc)/w)^2)
Plot	Qy (ROI 2)
y0	-0.00372 ± 0.0741
xc	-6.5977 ± 3.53296E-4
w	0.00424 ± 7.3863E-4
A	0.01097 ± 0.00179
Reduced Chi-Sqr	0.54987
R-Square (COD)	0.33178
Adj. R-Square	0.31561

Table S3

Model	Gauss
Equation	y=y0 + (A/(w*sqrt(pi/2)))*exp(-2*((x-xc)/w)^2)
Plot	Qx (ROI 3)
y0	0.14262 ± 0.14636
xc	-0.21576 ± 7.38454E-4
w	0.00148 ± 6.28345E-4
A	0.01134 ± 0.00431
Reduced Chi-Sqr	2.14221
R-Square (COD)	0.37838
Adj. R-Square	0.36111

Model	Gauss
Equation	y=y0 + (A/(w*sqrt(pi/2)))*exp(-2*((x-xc)/w)^2)
Plot	Qz (ROI3)
y0	0.2149 ± 0.18034
xc	-0.06347 ± 9.26492E-5
w	0.001 ± 1.88312E-4
A	0.01346 ± 0.00167
Reduced Chi-Sqr	3.89987
R-Square (COD)	0.45995
Adj. R-Square	0.44739

The values within the tables refers to the parameters of the 1D sections Qy-Qz for ROI 1 (Fig. 6), Qx-Qy for ROI 2 (Fig. 7) and Qx-Qz for ROI 3 (Fig. 8). For each fitting curve, the parameters have been brought to convergence by using a Gaussian function y= y0 + (A/(w*sqrt(pi/2)))*exp(-2*((x-xc)/w)^2), where:

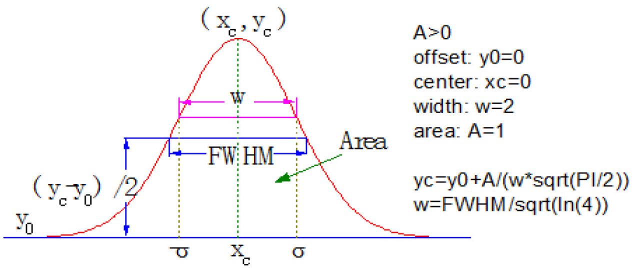

Figure S1 [Derived Parameters]

Sigma = w/2; // sigma.

FWHM = sqrt(2*ln(2))*w; // Full Width at Half Maximum

Height = A/(w*sqrt(pi/2)); // Height of the peak.

The Figures S2 and S3 below show 3D-RSMs with their relative 1D- and 2D projections of the Si 044 Bragg peak recorded at z = 3.5 µm of both pillars. The exact positions are indicated with white points in the SEM images S2(a) and S3(a) respectively. Beside to be in a different Q-position, the peaks within the ROI 3 split into two smaller peaks of different intensity. Such effect occurs exactly where there is a variation in the tilt distribution map, between 3.5 and 4.5 µm starting from the bottom of the pillars height (z) (Fig. S4). This phenomenon might be related to a dislocation or to a combination of more dislocations, introduced during the deep etching from the top to the bottom of the silicon crystal, in order to get the pillars height. However, as already mention in the text, for a detailed analysis of the defects, we would need a larger data set and/or perform a different and complementary technique such as HR-TEM, BCDI, XSW etc.

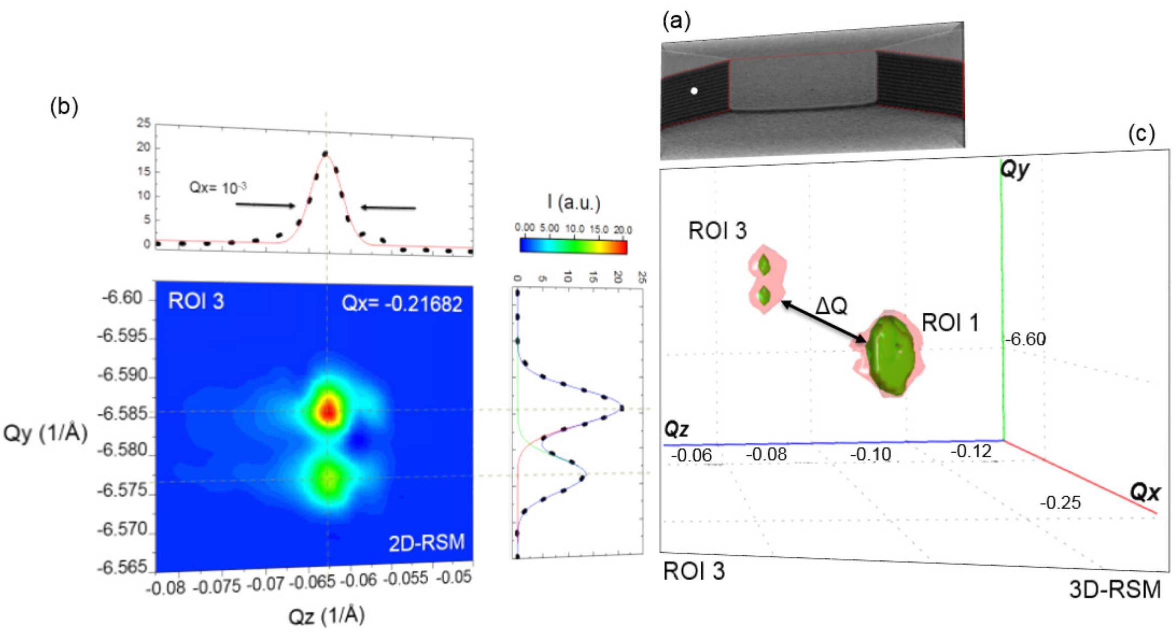

Model	Gauss
Equation	y=y0 + (A/(w*sqrt(pi/2)))*exp(-2*((x-xc)/w)^2)
Plot	Qy (ROI 3)
y0	-0.07639 ± 0.38532
xc	-6.59774 ± 1.56563E-4
w	0.00498 ± 3.30116E-4
A	0.16151 ± 0.01016
Reduced Chi-Sqr	14.4496
R-Square (COD)	0.76969
Adj. R-Square	0.7642

Model	Gauss
Equation	y=y0 + (A/(w*sqrt(pi/2)))*exp(-2*((x-xc)/w)^2)
Plot	Qz (ROI 3)
y0	0.09197 ± 1.06724
xc	-0.08719 ± 6.65245E-5
w	0.00188 ± 1.3558E-4
A	0.24908 ± 0.01616
Reduced Chi-Sqr	156.30762
R-Square (COD)	0.71187
Adj. R-Square	0.70611

Figure S2

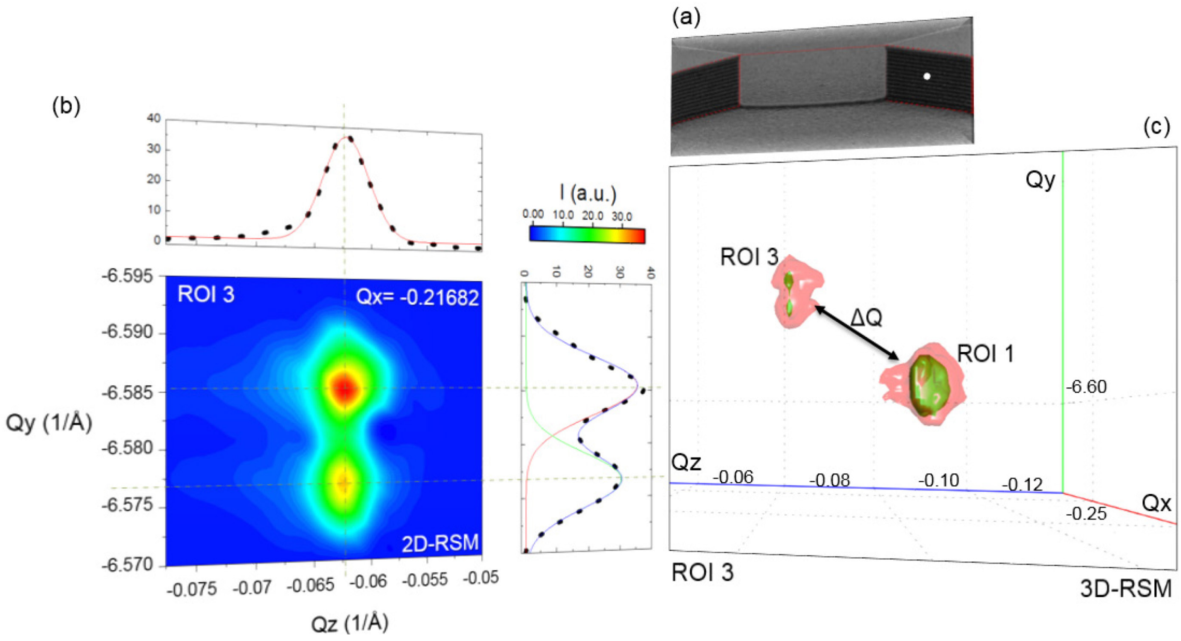

Model	Gauss
Equation	y=y0 + (A/(w*sqrt(pi/2)))*exp(-2*((x-xc)/w)^2)
Plot	Qy (ROI 3)
y0	-0.04208 ± 0.27161
xc	-6.5978 ± 1.38042E-4
w	0.00454 ± 2.89462E-4
A	0.11379 ± 0.00683
Reduced Chi-Sqr	7.39021
R-Square (COD)	0.78389
Adj. R-Square	0.77874

Model	Gaussian
Equation	y = y0 + A/(w*sqrt(pi/(4*ln(2)))) * exp(-4*ln(2)*(x-xc)^2/w^2)
Plot	Qz (ROI 3)
y0	0.07653 ± 0.80229
xc	-0.08697 ± 6.17776E-5
A	0.17784 ± 0.01168
w	0.00203 ± 1.50415E-4
Reduced Chi-Sqr	89.14612
R-Square (COD)	0.70765
Adj. R-Square	0.7018

Figure S3

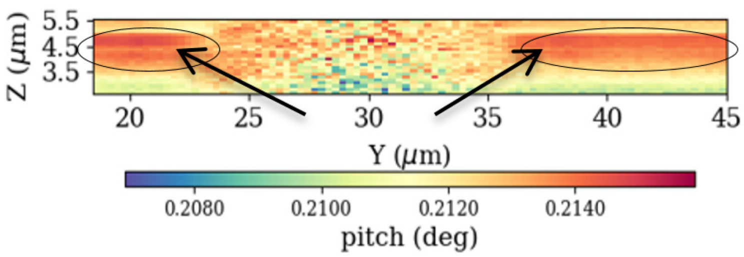

Figure S4 The black lines indicate the area where is present an important transition in the tilt distribution, bringing the Bragg peak to split into two smaller peaks of different intensity.

Given a coordinate system x, y, z with a vector $\boldsymbol{Q}$ and two angles (φ and θ), we can convert spherical coordinates into cartesian ones and vice versa.

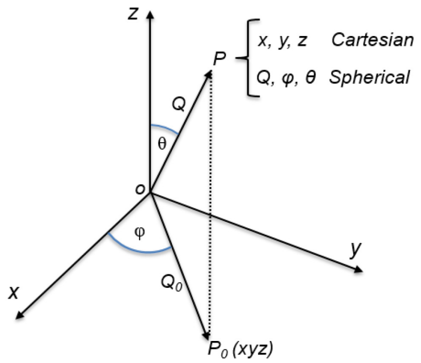

From cartesian to spherical we have:

$$Q = \sqrt{Q_x^2 + Q_y^2 + Q_z^2}$$

$$(\theta) = arccos\left[\frac{Qz}{\sqrt{Q_x^2 + Q_y^2 + Q_z^2}}\right] = arccos\left[\frac{Qz}{Q}\right]$$

$$(\varphi) = arctg\left[\frac{Qy}{Qx}\right]$$

From spherical to cartesian:

$Qx = Q \sin\theta \cos\varphi$

$Qy = Q \sin\theta \sin\varphi$

$Qz = Q \cos\theta$

Given two point A and B, of known coordinates, we want to calculate the direction angle (AB) expressed in centesimal degrees:

$$tg\ (AB) = \frac{x_B - x_A}{y_B - y_A}$$

$$\theta = \text{arctg}\left[\frac{x_B - x_A}{y_B - y_A}\right]$$

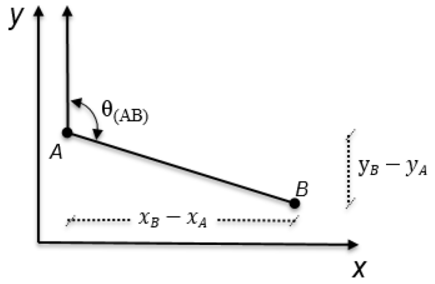

Supplement: Supplementary file 4 [file j-53-00058-sup4.pdf]
